# Prevalence and risk factors of sub-health and circadian rhythm disorder of cortisol, melatonin, and temperature among Chinese midwives

**DOI:** 10.3389/fpubh.2023.1142995

**Published:** 2023-02-15

**Authors:** Xiao-Qian Chen, Xiu-Min Jiang, Qing-Xiang Zheng, Hai-Wei Wang, Heng Xue, Yu-Qing Pan, Yan-Ping Liao, Xiao-Xia Gao

**Affiliations:** ^1^Fujian Maternity and Child Health Hospital College of Clinical Medicine for Obstetrics and Gynecology and Pediatrics, Fujian Medical University, Fuzhou, Fujian, China; ^2^Fujian Obstetrics and Gynecology Hospital, Fuzhou, Fujian, China; ^3^School of Nursing, Fujian Medical University, Fuzhou, Fujian, China

**Keywords:** circadian rhythm, cortisol, melatonin, midwife, sub-health

## Abstract

**Objective:**

This study aimed to explore the influencing factors of sub-health and circadian rhythm disorder among midwives and whether circadian rhythm disorder was associated with sub-health.

**Methods:**

A multi-center cross-sectional study was conducted among 91 Chinese midwives from six hospitals through cluster sampling. Data were collected by demographic questionnaire, Sub-Health Measurement Scale version 1.0, and circadian rhythm detection. Minnesota single and population mean cosine methods were used to analyze the rhythm of cortisol, melatonin, and temperature. Binary logistic regression, nomograph model, and forest plot were performed to identify variables associated with midwives' sub-health.

**Results:**

There were 65 midwives with sub-health and 61, 78, and 48 midwives with non-validation of circadian rhythms of cortisol, melatonin, and temperature among 91 midwives, respectively. Midwives' sub-health was significantly related to age, duration of exercise, weekly working hours, job satisfaction, cortisol rhythm, and melatonin rhythm. Based on these six factors, the nomogram was presented with significant predictive performance for sub-health. Furthermore, cortisol rhythm was significantly associated with physical, mental, and social sub-health, whereas melatonin rhythm was significantly correlated with physical sub-health.

**Conclusion:**

Sub-health and circadian rhythm disorder were generally common among midwives. Nurse administrators are supposed to pay attention and take measures to prevent sub-health and circadian rhythm disorder among midwives.

## 1. Introduction

Sub-health is an intermediate health status between health and illness, including physical, mental, and social symptoms. According to traditional Chinese medicine (TCM) guidelines released by the China Association of Chinese Medicine, sub-health is characterized as a decline in vitality, mental function, and the capacity for social adaptation, without clear disease in clinical diagnosis (1). The concept of sub-health, which is similar to chronic fatigue, has been widely accepted in Japan, Ghana, and Australia (2, 3). The sub-health prevalence took up 71.7% of nurses (4), 69.46% of Chinese adults (5), 55.9% of Chinese students (6), and 15.3% of rural migrants (7). People with sub-health status without recognizing and managing in time tend to suffer from chronic fatigue, headaches, dizziness, anxiety, depression, pain, and functional disorders of organ systems, which can impair quality of life and finally lead to a series of diseases (2, 8). In addition, there are complex causes of sub-health, such as lack of sleep time, lack of exercise, a heavy burden of study, smoking, drinking, and fewer friends (9). For medical staff, Spanish research showed that the influencing factors of medical staff's quality of life included work motivation, work burden, and manager's support (10). The lack of hospital resources, high workload, and organizational structure problems easily led to nurses' job burnout, affecting their health (11). Some studies from China also indicated that sub-health status among nurses was related to various factors such as demographic characteristics, mental status, occupation, social environment, unhealthy life, and work style (12, 13). Especially, it is essential to increase focus on the sub-health and its influencing factors in a hospital setting.

Midwives play an important role in ensuring and promoting the health of pregnant women and newborns (14). However, due to frequent rotating shifts, shortage of human resources, and high work stress, midwives easily suffer from sub-health (15, 16). Midwives reported moderate to severe levels of exhaustion on 22–50% of all shifts and rest days (17), and there were 59.3% of midwives with sub-health according to TCM guidelines (18). The United Nations Population Fund and the World Health Organization (WHO) have pointed out that there was a large shortage of midwives in the world (15). Furthermore, after the two-children policy in China in 2015, the number of older mothers and obstetric complications increased without a proportional increase in midwives (19). All make midwives bear more workload and suffer from sleep disorders, circadian rhythm disorder, fatigue, anxiety, and burnout, resulting in lower job satisfaction, higher turnover, and degradation in work quality (20, 21). Thus, it might be of great importance to early identify and prevent sub-health among midwives.

Circadian rhythm refers to the phenomenon of periodic life activities, presenting in almost all living organisms. Circadian rhythms are regulated by a central stimulator located in the hypothalamic suprachiasmatic nucleus (SCN) (22). SCN generates clock signals by receiving light exposure to control the sleep–wake cycle, body temperature, respiration, blood pressure, diet, hormone secretion, and energy metabolism (23, 24). So light exposure is an important zeitgeber for the circadian rhythm. Shift workers, who are exposed to nocturnal light, might suffer from an abnormal secretion of cortisol and melatonin or abnormal fluctuation of temperature, which can cause circadian rhythm disorder (25). Moreover, circadian rhythm disorder can increase the risk of mental and physical health problems (23). However, circadian rhythm cannot be directly evaluated in humans. Cortisol, melatonin, and core body temperature are considered circadian biomarkers (22). Cortisol is a steroid hormone released from the adrenal cortex into the circulation following activation of the hypothalamic–pituitary–adrenal (HPA) axis by stress and SCN (26). Cortisol can impact the body, mood, arousal, energy, metabolic processes, and immune and inflammatory system functioning (27). Meanwhile, melatonin is a hormone synthesized mainly in the pineal gland and secreted into the general circulation (28). It also plays a central role in the control of photoperiodic response (29). Melatonin is hydroxylated to 6-hydroxymelatonin in the liver, 90% of which is sulfated to 6-sulphatoxymelatonin (aMT6s), and then excretes in the urine (30). Urinary aMT6s is considered an easily measured indicator for the assessment of melatonin production. Besides, body temperature is a stable physiological variable, whose variation is correlated to the activity of the biological clock (22). Therefore, cortisol, melatonin, and body temperature can be used to indirectly assess the circadian rhythm.

Even though several studies have investigated the prevalence and risk factors of sub-health among nurses (4, 11, 13), studies examining midwives' sub-health were limited, and there is a lack of objective indicators to predict sub-health among midwives. In addition, it seems that circadian rhythm disorder may be a risk factor for sub-health. Therefore, the purpose of this study was to analyze the relationship between sub-health and circadian rhythm, including cortisol, melatonin, and temperature rhythm, and to explore the influencing factors of sub-health and circadian rhythm disorder among midwives.

## 2. Methods

### 2.1. Setting and sampling

This study was designed as a multi-center cross-sectional survey. Regarding the difficulties in sample collection, it was a prior study. A total of 100 midwives from six hospitals were selected through cluster sampling in the Fujian Province of China during January and March 2020. Subjects should meet the following inclusion criteria: (1) obtained professional qualification certificates; (2) more than 1 year of midwifery experience in the labor room; (3) no smoking and drinking coffee or tea 12 h before urine sample collection; (4) no taking any medicine 1 month before urine sample collection; and (5) volunteering to participate in this study. The exclusion criteria were as follows: (1) in menstrual and ovulation period; (2) in pregnancy and lactation; and (3) diagnosed as acute diseases or acute attack stage of chronic diseases according to their medical examination report.

### 2.2. Measures

#### 2.2.1. Demographic questionnaire

The demographic questionnaire was designed by the researchers based on a literature review, including age, educational level, monthly income, marital status, diet and exercise habits, adverse life events in the past year, body mass index (BMI), nature and rank of the hospital, years of midwifery experience, professional rank, employment type, shift work or not, weekly working hours, workload, occupational injuries in the past year, and job satisfaction. Adverse life events include eight kinds of problems, such as accidents, marital barriers, and children's problems. According to the Chinese standard, BMI is classified into four categories: thin, BMI < 18.5; normal, 18.5 < BMI < 23.9; overweight, 24.0 < BMI < 27.9; and obesity, BMI>28.0. The workload was defined as the ratio of an annual delivery number to a midwife number in their hospitals. WHO recommends each midwife be responsible for 175 births per year (31). Therefore, this study defined a ratio >175 per year as a high workload. Otherwise, it was a low workload. Occupational injuries included: (1) acupuncture injuries; (2) cutting injuries; (3) splashing of blood into eyes, skin, and mucosa; and (4) threatened, abused, or beaten by childbirth women or their family members. Job satisfaction was divided into “yes” and “no”. All continuous variables were divided into categorical data.

#### 2.2.2. Sub-health measurement scale version 1.0 (SHMS V1.0)

The SHMS V1.0, developed by Jun Xu et al. (32), was used to assess the sub-health state of midwives. The scale consists of 39 items with three dimensions: physical sub-health (14 items), mental sub-health (12 items), social sub-health (nine items), and other four sub-health overall assessment items. The scoring pattern for each item is a 5-point Likert scale (1 = never, 2 = occasionally, 3 = sometimes, 4 = often, and 5 = routinely). The total score for SHMS V1.0 domains was transformed to a range of 0 to 100, with the lower scores representing higher sub-health severity. SHMS V1.0 is a valid and reliable tool in various ethnic groups, with Cronbach's alpha and split-half reliability coefficients of 0.917 and 0.831, respectively (32). According to the demarcation score of SHMS V1.0 among midwives in the previous study, midwives with SHMS V1.0 scores of < 65 were classified as sub-health (33). Otherwise, it was considered as health.

### 2.3. Circadian rhythm detection

The cortisol, melatonin, and body temperature were detected to assess circadian rhythm. To ensure that the participants slept sufficiently, participants were asked to record armpit temperature and collect urine samples on two consecutive day shifts after their days off. The armpit temperature of the subjects was monitored by a mercury thermometer at 06:00, 08:00, 12:00, 16:00, 20:00, and 24:00. A 6-ml sample of each subject's midstream urine was collected at 07:00, 11:00, 19:00, 23:00. Thus, 12-time armpit temperatures and eight urine samples were obtained from every subject over 48 h period of sampling. All urine samples were centrifuged at 3,000 rpm for 8 min, then ~2 × 2 ml of urine was separated and stored in a refrigerator at −80°C for further testing. The subject's aMT6s level was tested by enzyme-linked immunosorbent assay (ELISA) (the brand of ELISA Kit for Melatonin: Cloud-Clone Corp, Catalog number: CEA908. Testing Instrument: Biotek Elx800). The urinary cortisol was assayed by the chemiluminescence method (The brand of Access Cortisol: Beckman Coulter, Catalog number: 33600. Testing Instrument: Access).

Minnesota single cosine method and Minnesota population mean cosine method were used to analyze the circadian rhythm of cortisol, melatonin, and temperature. Using the Halberg cosine method software package, a package for chronobiological analysis, four rhythmic parameters were determined by the least square method (29): mesor (mean level, a time series of 48 h), amplitude (the difference between the maximum value of the cosine function and the mesor), acrophase (time of peak), and *P*-value (*P* < 0.05 indicates that the circadian rhythm is normal; *P* > 0.05 indicates that the circadian rhythm is disordered). Minnesota population mean cosine method was used to fit the circadian rhythm of all the subjects to reflect the average characteristics of the group's circadian rhythm, and the meaning of the *P*-value was the same as that of the Minnesota single cosine method. In this study, circadian rhythm disorder meant non-validation of the circadian rhythm of cortisol, melatonin, and temperature on two consecutive day shifts by cosinor.

### 2.4. Data collection

Data were obtained by questionnaire surveys and urine sample collection. Informed consent was obtained from the nursing department of six sample hospitals before the investigation. The six head nurses from the delivery room of sample hospitals were trained and then conducted the investigation. The six trained head nurses chose subjects according to the inclusion criteria and then explained the research purpose, filling method of the questionnaire, methods and precautions of body temperature measurement, and urine specimen collection of subjects. After obtaining the informed consent of the subjects, the head nurses distributed the number, questionnaire, thermometer, temperature record sheet, sterile test tube, and urine cup to the subjects. The subjects were required to wrap the urine samples in black plastic bags and temporarily store those in a 4°C refrigerator. Researchers visited each sample collection hospital once or twice a day and sent the urine samples with ice packs to the laboratory for unified detection until all urine samples were collected. At the same time, questionnaires were collected by head nurses and sent to the researcher. The subjects who did not complete the collection of all urine specimens were excluded from the study.

### 2.5. Statistical analyses

All data were analyzed using the IBM SPSS, version 25.0, and “rms” and “forest plot” packages in R statistical software, version 4.0.5. Descriptive statistics including frequency, percentage, means (M), and standard deviations (SD) were used to describe continuous and categorical variables. The comparative analysis of the categorical data was performed using the Chi-square test or Fisher's exact test. An unconditional binary logistic regression model was applied to identify factors influencing midwives' sub-health. Based on multivariable logistic regression analysis, a forest plot was generated to present the risk factors and odds ratio (OR), 95% confidence interval (95%CI) for correlations with the sub-health, and to build a more accurate and reliable sub-health prediction model, a nomogram was constructed according to the parameters of the statistical regression model. This study used the concordance index (C index) to evaluate the discriminative ability of the nomograph model. All tests were two-sided and *p*-values < 0.05 were regarded as statistically significant.

## 3. Results

### 3.1. Participant characteristics

A total of 100 midwives were eligible and invited to participate in this study, of which 91 completed all questionnaires and circadian rhythm detection (effective recovery rate was 91%). Nine subjects without 8 times urine specimens collected were invalid. For demographic and life-related characteristics, the mean age of midwives was 30.74 years (SD = 5.746) and 40.7% of midwives had a junior college degree. Most midwives had a monthly income of < 9,000 RMB (1 RMB = 0.1446 USD) and 61.5% of midwives were married. About half of the midwives often had irregular meals (42.9%) and did exercise ranging from one to two times per week (60.4%). One-third of midwives suffered from more than one kind of adverse life event in the past year (25.3%). There were 27.5% of midwives with abnormal BMI (thin/overweight/obesity). For work-related characteristics, midwives came from tertiary (81.3%) and secondary (18.7%) levels of hospital and 62.6% of hospitals were general hospital. Approximately half of the midwives had < 5 years of midwifery and senior professional rank. Employment types included formal employees (41.8%) and contract employees (58.2%). The majority of midwives had shift work with a high workload and 49.5% of midwives worked more than 40 h weekly. Half of the midwives had experienced more than one kind of occupational injury in the past year and 39.6% of participants reported no job satisfaction (Table 1).

**Table 1:** Differences of midwives' sub-health between/among variables (*n* = 91).

**Variables**	***N*** **(%)**		***X* ^2^**	***P***
	**Health**	**Sub-health**		
**Age (year)**			2.23	0.329
≤ 25	7 (36.84)	12 (63.16)		
26–35	16 (30.19)	37 (69.81)		
≥36	3 (15.79)	16 (84.21)		
**Educational level**			0.55	0.458
Junior college degree	9 (24.32)	28 (75.68)		
Bachelor degree and above	17 (31.48)	37 (68.52)		
**Monthly income (yuan)**			0.58	0.748
< 6,000	12 (27.27)	32 (72.73)		
6,000–8,999	4 (23.53)	13 (76.47)		
≥9,000	10 (33.33)	20 (66.67)		
**Marital status**			3.64	0.056
Unmarried	14 (40.00)	21 (60.00)		
Married	12 (21.43)	44 (78.57)		
**Frequency of irregular meals**			2.17	0.141
Sometimes (1–2 days/week)	18 (34.62)	34 (65.38)		
Often (≥3 days/week)	8 (20.51)	31 (79.49)		
**Frequency of exercise**			6.30	**0.043**
No	4 (14.29)	24 (85.71)		
Sometimes (1–2 days/week)	21 (38.18)	34 (61.82)		
Often (≥3 days/week)	1 (12.50)	7 (85.50)		
**Duration of each exercise**			5.60	0.061
No	3 (11.54)	23 (88.46)		
< 30 min	16 (38.10)	26 (61.90)		
≥30 min	7 (30.43)	16 (69.57)		
**How many types of adverse life events in the past year have you experienced?**			1.66	0.485a
0	22 (32.35)	46 (67.65)		
1	3 (20.00)	12 (80.00)		
≥2	1 (12.50)	7 (87.50)		
**BMI**			2.52	0.274a
Thin	2 (13.33)	13 (86.67)		
Normal	22 (33.33)	44 (66.67)		
Overweight or obesity	2 (20.00)	8 (80.00)		
**Hospital rank**			0.01	1.000
Tertiary	21 (28.38)	53 (71.62)		
	**Health**	**Sub-health**		
Secondary	5 (29.41)	12 (70.59)		
**Hospital nature**			1.202	0.273
General hospital	14 (24.56)	43 (75.44)		
Specialized hospital	12 (35.29)	22 (64.71)		
**Years of midwifery experience (year)**			2.51	0.479a
≤ 5	12 (29.27)	29 (70.73)		
6–10	9 (39.13)	14 (60.87)		
11–20	4 (18.18)	18 (81.82)		
≥21	1 (20.00)	4 (80.00)		
**Professional rank**			0.65	0.723
Junior nurse	6 (30.00)	14 (70.00)		
Senior nurse	17 (30.36)	39 (69.64)		
Assistant advanced nurse or above	3 (20.00)	12 (80.00)		
**Employment type**			0.29	0.591
Formal employees	12 (31.58)	26 (68.42)		
Contract employees	14 (26.42)	39 (73.58)		
**Shift work**			0.20	1.000
No	2 (22.22)	7 (77.78)		
Yes	24 (29.27)	58 (70.73)		
**Weekly working hours (hour)**			7.39	**0.007**
< 40	19 (41.30)	27 (58.70)		
≥40	7 (15.56)	38 (84.44)		
**Workload**			2.85	0.092
Low (≤ 175)	7 (18.92)	30 (81.08)		
High (>175)	19 (35.19)	35 (64.81)		
**How many types of occupational injuries in the past year have you experienced?**			2.750	0.253
0	18 (35.29)	33 (64.71)		
1	4 (23.53)	13 (76.47)		
≥2	4 (17.39)	19 (82.61)		
**Job satisfaction**			11.59	**0.001**
Yes	23 (41.82)	32 (58.18)		
No	3 (8.33)	33 (91.67)		
**Cortisol rhythm**			17.31	**< 0.001**
Normal	17 (56.67)	13 (43.33)		
Disorder	9 (14.75)	52 (85.25)		
**Melatonin rhythm**			4.75	**0.045**
Normal	7 (53.85)	6 (46.15)		
	**Health**	**Sub-health**		
Disorder	19 (24.36)	59 (75.64)		
**Body temperature rhythm**			0.64	0.426
Normal	14 (32.56)	29 (67.44)		
Disorder	12 (25.00)	36 (75.00)		

BMI, body mass index. χ^2^, the statistics of chi-square test or Fisher's exact test. Bold values indicates that the *p* value is less than 0.05, indicating statistical significance.

### 3.2. The prevalence of sub-health and circadian rhythm disorder among midwives

The SHMS V1.0 score of 65 midwives was < 65, indicating that 65 (71.4%) midwives were under sub-health status. Results of Minnesota single cosine analysis showed that 61 (67.0%) midwives had non-validation of the circadian rhythm of cortisol, with 52 midwives under sub-health. There were 78 (85.7%) midwives with non-validation of circadian rhythm of melatonin, of which 59 midwives were under sub-health. In addition, 48 (52.7%) midwives had non-validation of circadian rhythm of temperature, of which 36 midwives were under sub-health. Moreover, the results of the Minnesota population mean cosine analysis indicated that the group's circadian rhythms of cortisol, melatonin, and temperature were all disordered (Table 2).

**Table 2:** Minnesota population mean cosine analysis of group's circadian rhythm (*n* = 91).

**Items**	**Mesor**	**Amplitude**	**Acrophase**	***P***
Cortisol biorhythm	11.12	−3.53	0.64	0.280
Melatonin biorhythm	10.77	0.51	0.79	0.141
Temperature biorhythm	36.38	−0.07	0.12	0.300

### 3.3. Factors influencing circadian rhythm disorder among midwives

Demographic data, work, and life-related factors were all entered in the logistic regression model for Wald back analysis. The ordered categorical variables were assigned and then enter logistic regression analysis as continuous variables. After adjusted analysis, the results indicated that there were different influencing factors of cortisol, melatonin, and temperature biorhythm disorder. Significant factors influencing cortisol biorhythm disorder included age (OR = 9.11, 95% CI = 1.38 to 59.99, *P* = 0.022), workload (OR = 9.19, 95 % CI = 1.70 to 49.72, *P* = 0.010), and job satisfaction (OR = 7.00, 95% CI = 1.94 to 25.25, *P* = 0.003). However, age, workload, and job satisfaction had no significant impact on melatonin and temperature biorhythm disorder. Melatonin biorhythm disorder was only related to the frequency of exercise (OR = 0.20, 95% CI = 0.04 to 0.90, *P* = 0.037). The exercise was not an influencing factor of cortisol and temperature biorhythm disorder. For temperature biorhythm disorder, it was only related to employment type (OR = 4.59, 95% CI = 1.88 to 11.23, *P* = 0.001). However, employment type did not influence cortisol and melatonin biorhythm disorder. Furthermore, educational level, monthly income, marital status, frequency of irregular meals, duration of exercise, irregular meals, adverse life events in the past year, BMI, nature and rank of the hospital, years of midwifery experience, professional rank, shift work or not, weekly working hours, and occupational injuries in the past year were all not significant influencing factors of cortisol, melatonin, and temperature biorhythm disorder (Table 3).

**Table 3:** Multivariable logistic regression analysis for influence factors of biorhythm disorder (*n* = 91).

**Items**	**Variables**	**β**	***SE***	***Wald***	***P***	***OR***	**95%** ***CI*** **of** ***OR***	
							**Lower bound**	**Upper bound**
Cortisol biorhythm	Age (year)	2.209	0.962	5.280	0.022	9.11	1.38	59.99
	Workload^†^	2.218	0.862	6.626	0.010	9.19	1.70	49.72
	Job satisfaction^‡^	1.946	0.654	8.846	0.003	7.00	1.94	25.25
	Constant	−2.614	1.303	4.024	0.045	0.07		
Melatonin biorhythm	Frequency of exercise	−1.626	0.778	4.366	0.037	0.20	0.04	0.90
	Constant	2.089	0.985	4.494	0.034	8.08		
Temperature biorhythm	Employment type^§^	1.523	0.457	11.138	0.001	4.59	1.88	11.23
	Constant	−0.773	0.349	4.908	0.027	0.46		

^†^The reference group was “low workload”, ^‡^The reference group was “Yes”, ^§^The reference group was “Formal employees”.

95% CI, 95% confidence intervals.

### 3.4. Influence factors of midwives' sub-health

**Figure 1:**
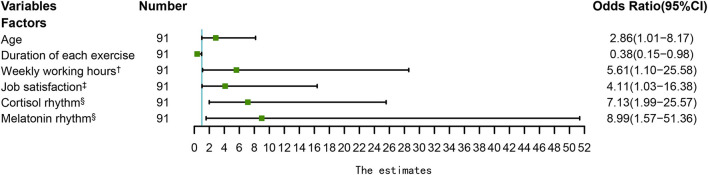
The forest plot of sub-health influence factors among midwives. †: The reference group was “ < 40”, ‡: The reference group was “satisfied”, §: The reference group was “Disorder”. (Model adjusted for educational level, monthly income, marital status, frequency of irregular meals, frequency of exercise, adverse life events in the past year, BMI, nature and rank of the hospital, years of midwifery experience, professional rank, employment type, shift work or not, workload, occupational injuries in the past year and temperature circadian rhythm.).

Univariable analysis indicated that midwives' sub-health was significantly related to the frequency of exercise, weekly working hours, job satisfaction, cortisol rhythm, and melatonin rhythm, while temperature circadian rhythm was not related to midwives' sub-health (Table 1). The forest plot based on multivariable logistic regression analysis showed that significant factors influencing sub-health included age (OR = 2.86, 95% CI = 1.01 to 8.17, *P* = 0.049), duration of exercise (OR = 0.38, 95% CI = 0.15 to 0.98, *P* = 0.045), weekly working hours (OR = 5.61, 95% CI = 1.10 to 25.58, *P* = 0.038), job satisfaction (OR = 4.11, 95% CI = 1.03 to 16.38, *P* = 0.045), cortisol rhythm (OR = 7.13, 95% CI = 1.99 to 25.57, *P* = 0.003), and melatonin rhythm (OR = 8.99, 95% CI = 1.57 to 51.36, *P* = 0.014) in the final regression model (Figure 1). However, educational level, monthly income, marital status, frequency of irregular meals, frequency of exercise, adverse life events in the past year, BMI, nature and rank of the hospital, years of midwifery experience, professional rank, employment type, shift work or not, workload, occupational injuries in the past year, and temperature circadian rhythm had no significant influence on midwives' sub-health.

### 3.5. Nomograph model for midwives' sub-health

**Figure 2:**
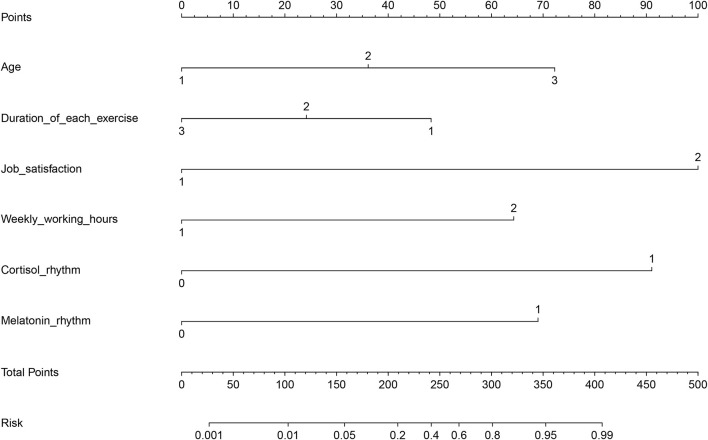
Nomogram for the predictors of midwives' sub-health. C index: 0.89, 95% CI of C index: 0.82 to 0.96. In the nomogram, an individual subject's value is located on each variable axis, and a line is drawn upward to determine the number of points received for each variable value. The sum of these numbers is located on the Total Points axis to determine the risk of sub-health.

According to the results of multivariable logistic regression analysis, we performed a nomogram to predict the possibility of sub-health among midwives. The score assigned to each factor was proportional to its risk contribution to sub-health (Figure 2). The C index of the nomogram model was 0.89 (95% CI 0.82 to 0.96), which demonstrated good agreement between predictors and the actual outcome. For example, older age, less exercise time, longer working hours, dissatisfaction with their work, and cortisol and melatonin circadian rhythm disorder could accurately predict sub-health status among midwives.

### 3.6. Associations between circadian rhythm and sub-health among midwives

The multivariable logistic regression model indicated that cortisol and melatonin rhythm disorder were predictors of midwives' sub-health. Therefore, the study continued to explore the relationship between cortisol, melatonin rhythm, and each dimension of sub-health, including physical, mental, and social sub-health. The results showed that there were 65, 62, and 59 midwives with physical, mental, and social sub-health, respectively. For cortisol circadian rhythm, 50, 46, and 45 midwives had physical, mental, and social sub-health among 61 midwives with cortisol rhythm disorder, respectively. In addition, cortisol rhythm was significantly related to all dimensions of sub-health, including physical (c^2^ = 10.07, *P* = 0.002), mental (c^2^ = 4.51, *P* = 0.034), and social sub-health (c^2^ = 6.48, *P* = 0.011). Among 78 midwives with melatonin rhythm disorder, there were 59, 55, and 53 midwives with physical, mental, and social sub-health. Moreover, melatonin rhythm was only related to physical sub-health (c^2^ = 4.75, *P* = 0.045), without relating to mental and social sub-health (Table 4).

**Table 4:** Relationship between circadian rhythm and sub-health in midwives (*n* = 91).

**Item**		***N*** **(%)**					
		**Physical health**	**Physical sub-health**	**Psychological health**	**Psychological sub-health**	**Social health**	**Social sub-health**
Cortisol	Normal	15 (50.00)	15 (50.00)	14 (46.67)	16 (53.33)	16 (53.33)	14 (46.67)
	Disorder	11 (28.38)	50 (81.97)	15 (24.59)	46 (75.41)	16 (26.23)	45 (73.77)
*X* ^2^		10.07		4.51		6.48	
*P*		**0.002**		**0.034**		**0.011**	
Melatonin	Normal	7 (53.85)	6 (46.15)	6 (46.15)	7 (53.85)	7 (53.85)	6 (46.15)
	Disorder	19 (24.36)	59 (75.64)	23 (29.49)	55 (70.51)	25 (32.05)	53 (67.95)
*X* ^2^		4.75		1.43		2.32	
*P*		**0.045**		0.334		0.208	

Bold indicates that the p value is less than 0.05, indicating statistical significance.

## 4. Discussion

The results of the study revealed that most midwives were under sub-health status and had circadian rhythm disorder of cortisol, melatonin, and temperature. The influencing factors of cortisol biorhythm disorder among midwives were age, workload, and job satisfaction; melatonin biorhythm disorder was related to the frequency of exercise; employment type influenced temperature biorhythm disorder. Predictors of midwives' sub-health were age, exercise, weekly working hours, job satisfaction, cortisol rhythm, and melatonin rhythm. Furthermore, cortisol rhythm was significantly associated with physical, mental, and social sub-health, and there was a significant correlation between melatonin rhythm and physical sub-health.

This study suggested that midwives had a high incidence of sub-health. This might be because the midwifery profession requires a lot of commitment to working with and caring for patients. Midwives often work excessive hours or have overnight shifts due to heavy workloads and a shortage of staff (34). In addition, midwives also need high emotional involvement to deal with women's concerns and anxieties during the intrapartum period and to instill their confidence in labor (35). Furthermore, midwives as women appear to be more vulnerable to exhaustion because of physiological characteristics, social struggle, and household responsibilities (36, 37). Those all can take a huge physical, mental, and social toll on midwives (38).

This study also found that most midwives had cortisol, melatonin, and temperature biorhythm disorders. Human beings have developed their biochemical and physiological processes such as cell cycle, apoptosis, or hormonal secretion around the 24-h rhythm marked by Earth's rotation around the Sun, which is organized by exposure to light (39). Night shift contributes to circadian disruption affecting hormonal systems regulating metabolism and stress responses because of exposure to light at night, which could dysregulate the HPA axis (40). The study revealed that long-term rotating shifts work have disrupted the cortisol, melatonin, and temperature circadian rhythms of midwives, though midwives in the study all recorded armpit temperature and collected urine samples during the day shift to meet the 24-h light cycle. Disruption of circadian rhythms can result in physical and mental disorders, the same as symptoms of sub-health (25), and even lead to metabolic diseases, cardiovascular diseases, and mood disturbances (41). These can increase the risk of making mistakes during the shift for midwives, which may cause a loss in the quality of care (39). Therefore, nursing managers should pay more attention to the sub-health or circadian rhythm disorder of midwives.

The results of logistic regression analysis and nomogram in the study indicated that older midwives had less exercise time, worked longer hours, were dissatisfied with their work, had cortisol and melatonin rhythm disorders, and were prone to sub-health status. Age is an irresistible factor in physiological function change. So, the older the midwives were, the higher the incidence of sub-health they had. Job satisfaction may promote work motivation and make midwives feel less occupational stress, which contributes to relieving fatigue, and then it could decrease the risk of sub-health (42). More working hours may lead to work–recreation imbalance conditions, which were associated with increased risk for sub-health. In addition, work–recreation balance conditions seem to be accurate behavioral indicia of a healthy lifestyle (43). Regarding frequency and duration of exercise, the results of univariable analysis and multivariable logistic regression analysis were incompatible. However, they all pointed to the undeniable benefits of exercise. More frequent and longer periods of exercise were related to a lower risk of sub-health, which was aligned with the study of Yu et al. (44), which identified that lack of exercise, as a lifestyle factor, strongly contributed to chronic fatigue.

With regard to circadian rhythm disorder, cortisol rhythm was associated with physical, mental, and social sub-health among midwives. Cortisol is one of the keys to wellbeing and plays a vital role in balancing physiological changes, mental health, and behavior of humans. The desynchronization of cortisol influences sleep quality and quantity and immune and inflammatory system functioning (23), which are associated with physical sub-health. When individuals experience cortisol rhythm disruption, there is a functional imbalance between the ventral anterior cingulate cortex and the amygdala, which are the emotional center of the brain (45). So cortisol rhythm disruption could lead to mood disorders (46), which is related to mental sub-health. In addition, cortisol status may influence overall health as well as essential work skills, such as attention (47). Therefore, it seems that midwives with cortisol rhythm disorder may have bad work performance, increasing work stress, and tension in interpersonal relationships, leading to social sub-health. For melatonin rhythm, it was only significantly correlated to physical sub-health. Melatonin provides the coordination of physiological functions including the sleep–wake cycle, diet intake, hormone secretion, and metabolism. Changes in light intensity, duration, and spectral quality at a certain time, which often occur in night shift workers who are exposed to light during night-time hours, acutely suppressed the secretion of melatonin and can cause various diseases (25).

The findings of this study contribute to the limited research on the status of midwives' sub-health and circadian rhythm and explore the influence factors of midwives' sub-health. Moreover, it was a multi-hospital study to identify the associations between sub-health and circadian rhythm. However, there are several limitations to this study. First, the sample size of this survey was small because it was very difficult for the researcher to collect eight or more urine samples from more subjects on 2 consecutive days, but it was a prior study to explore the association of midwives' sub-health with circadian rhythm. Second, a cross-sectional study design might limit the power of causal relationships between the predictors and sub-health. Therefore, more prospective studies with large sample sizes are needed to identify the relationship between midwives' sub-health and its associated factors.

Based on the aforementioned evidence of this study, it is important to pay attention to timely assess and improve the sub-health and biological rhythm disorder of midwives. First, nursing managers had better provide sufficient resources and establish a reasonable incentive mechanism, which is conducive to improving the job satisfaction of midwives (2). Second, it is of great significance to set up a scientific scheduling mode and arrange working hours reasonably to alleviate sleep disorders and melatonin rhythm disruption. Third, nursing managers are supposed to give positive expectations and support to midwives, timely assess and ease the mental problems of midwives to avoid adverse stress reactions, which can affect the secretion of cortisol. For individuals, midwives should improve their unhealthy lifestyles, keeping proper exercise and enough sleep to promote health. At the same time, midwives should learn to coordinate the relationship between work and family.

## 5. Conclusion

Most midwives were under sub-health status and circadian rhythm disorder of cortisol, melatonin, and temperature. Age, duration of exercise, weekly working hours, job satisfaction, cortisol, and melatonin rhythm disorder had a significant influence on sub-health among midwives. Therefore, the findings of this study highlight the importance of providing strategies to reduce and prevent midwives' sub-health and circadian rhythm disorder.

## Data availability statement

The raw data supporting the conclusions of this article will be made available by the authors, without undue reservation.

## Ethics statement

The studies involving human participants were reviewed and approved by the Ethical Committee of Fujian Maternal and Child Health Hospital (No: 2018-206). The patients/participants provided their written informed consent to participate in this study.

## Author contributions

X-QC: study design and conduct, data collection and analysis, manuscript drafting, and manuscript revision. X-XG and Y-PL: data collection. Y-QP: data collection and specimen detection. HX: specimen detection and data analysis. H-WW: data interpretation and manuscript revision. Q-XZ: specimen detection and manuscript revision. X-MJ: study design, data interpretation, and manuscript revision.
